# Mesenchymal Stem Cell-Derived Exosomes and MicroRNAs in Cartilage Regeneration: Biogenesis, Efficacy, miRNA Enrichment and Delivery

**DOI:** 10.3390/ph14111093

**Published:** 2021-10-28

**Authors:** Jhi Biau Foo, Qi Hao Looi, Chee Wun How, Sau Har Lee, Maimonah Eissa Al-Masawa, Pei Pei Chong, Jia Xian Law

**Affiliations:** 1 School of Pharmacy, Faculty of Health and Medical Sciences, Taylor’s University, Subang Jaya 47500, Malaysia; JhiBiau.Foo@taylors.edu.my; 2 Centre for Drug Discovery and Molecular Pharmacology (CDDMP), Faculty of Health and Medical Sciences, Taylor’s University, Subang Jaya 47500, Malaysia; SauHar.Lee@taylors.edu.my; 3 My Cytohealth Sdn. Bhd., D353a, Menara Suezcap 1, KL Gateway, no. 2, Jalan Kerinchi, Gerbang Kerinchi Lestari, Kuala Lumpur 59200, Malaysia; dr.daniellooi@cytoholdings.com; 4 National Orthopaedic Centre of Excellence in Research and Learning (NOCERAL), Department of Orthopaedic Surgery, Faculty of Medicine, University of Malaya, Kuala Lumpur 50603, Malaysia; 5 School of Pharmacy, Monash University Malaysia, Bandar Sunway 47500, Malaysia; how.cheewun@monash.edu; 6 Faculty of Health and Medical Sciences, School of Biosciences, Taylor’s University, Subang Jaya 47500, Malaysia; PeiPei.Chong@taylors.edu.my; 7 Centre for Tissue Engineering and Regenerative Medicine, Faculty of Medicine, Universiti Kebangsaan Malaysia Medical Centre, Jalan Yaacob Latif, Kuala Lumpur 56000, Malaysia; maimonah.almasawa@gmail.com

**Keywords:** exosomes, cartilage, osteoarthritis, microRNA, chondrocyte

## Abstract

Exosomes are the small extracellular vesicles secreted by cells for intercellular communication. Exosomes are rich in therapeutic cargos such as microRNA (miRNA), long non-coding RNA (lncRNA), small interfering RNA (siRNA), DNA, protein, and lipids. Recently, many studies have focused on miRNAs as a promising therapeutic factor to support cartilage regeneration. Exosomes are known to contain a substantial amount of a variety of miRNAs. miRNAs regulate the post-transcriptional gene expression by base-pairing with the target messenger RNA (mRNA), leading to gene silencing. Several exosomal miRNAs have been found to play a role in cartilage regeneration by promoting chondrocyte proliferation and matrix secretion, reducing scar tissue formation, and subsiding inflammation. The exosomal miRNA cargo can be modulated using techniques such as cell transfection and priming as well as post-secretion modifications to upregulate specific miRNAs to enhance the therapeutic effect. Exosomes are delivered to the joints through direct injection or via encapsulation within a scaffold for sustained release. To date, exosome therapy for cartilage injuries has yet to be optimized as the ideal cell source for exosomes, and the dose and method of delivery have yet to be identified. More importantly, a deeper understanding of the role of exosomal miRNAs in cartilage repair is paramount for the development of more effective exosome therapy.

## 1. Introduction

Cartilage damage is very common and is one of the hallmarks of degenerative joint disorders such as osteoarthritis (OA) [1]. Age, gender (female), obesity, history of knee injury, joint overuse, joint abnormality, and genetics are known to increase the risk of OA [2,3]. Relying on cartilage self-repair is insufficient to restore the structure and function of the degenerated tissue as cartilage tissue has very poor regeneration potential [4]. Unlike most tissues, cartilage is avascular, alymphatic, and anueral and has low cellularity, with sparsely distributed chondrocytes embedded within the dense extracellular matrix (ECM) [5]. Thus, early intervention is critical for the management of OA. Generally, OA management can be categorized into non-pharmacological and pharmacological management modalities. Regardless of the treatment approaches, current interventions aim to prevent further damage and achieve symptoms control. Joint replacement surgery is typically prescribed when the damage is very extensive and severely affects the patient’s quality of life [6].

Regenerative medicine is a new approach introduced in the last two decades to promote the regeneration of damaged cartilage. This approach is unique as it can stimulate cartilage regeneration, which cannot be achieved with conventional treatments. In recent years, instead of cells, more attention has been given to the paracrine factors secreted by cells. In particular, exosomes, a type of small extracellular vesicle (EV) secreted by cells for intercellular communication, have become the focus of recent studies. Exosomes are rich in proteins, lipids, and nucleic acids and have been documented to promote cartilage regeneration [7,8]. Previously, a battery of miRNAs was found to be involved in cartilage regeneration [9,10,11]. MicroRNAs (miRNAs) are short single-stranded non-coding RNAs, approximately 22 nucleotides in length, that play important roles in post-transcriptional gene regulation [12]. In this review, we summarize the roles of exosomal miRNAs in cartilage regeneration. In addition, we also discuss the techniques used to modify the miRNA cargos of exosomes.

## 2. Cartilage Damage and Osteoarthritis

Finding a cure for cartilage damage and injury is still a major challenge despite significant advancements in the field in recent years. The difficulty stems, in part, from the unique structure of cartilage, which lacks vascular, neural, and lymphatic supplies and relies totally on diffusion for the exchange of nutrients and metabolites [13,14]. In addition, chondrocytes, which are the primary cell type in cartilage tissue, are scarce, making up only 5–10% of total cartilage mass [14], and are confined within the ECM, which possibly limits their ability to migrate to injured areas [15]. Thus, damaged cartilage has limited regenerative potential, and, in many instances, damaged cartilage is replaced with fibrocartilage or scar tissue with poorer functional and structural properties [4]. Within the developing cartilage, the primary role of chondrocytes is to proliferate and produce ECM constituents [16,17]. Astonishingly, chondrocytes can precisely synthesize ECM macromolecules such as type II collagen, proteoglycans (primarily aggrecan), glycosaminoglycans (GAGs) such as hyaluronan, and other non-collagenous constituents essential for the tissue [18]. Eventually, chondrocytes and their synthesized ECM form a highly organized structure that supports cartilage functionality [19,20].

In adults, chondrocytes stop proliferating but persist in maintaining cartilage tissue by continuously remodeling the macromolecular framework of the matrix through enzymatic degradation of matrix constituents, substituting them with newly synthesized macromolecules. The balance in the anabolic and catabolic activities is critical to maintaining cartilage homeostasis [19,21]. Growth factors play important roles in maintaining the chondrocyte differentiation state and controlling the production of ECM components [22]. The ECM, in turn, has a great influence on chondrocyte functionality. The ECM is known to protect the chondrocytes, influence the nutrient and metabolite diffusion, and modulate chondrocyte migration and attachment. In addition, the ECM also interacts with chondrocytes through cell receptors and binding epitopes to regulate cell proliferation, differentiation, and secretion of growth factors and cytokines [23]. Damage to the ECM will disrupt the dynamic interaction between the cartilage matrix and chondrocytes, thereby adversely affecting the metabolic activity and phenotypic stability of the residing chondrocytes. Consequently, alteration of chondrocyte functions leads to aberrant matrix production, culminating in an endless cycle of disturbance to cartilage homeostasis [24].

Cartilage degeneration is the hallmark of OA, the most common degenerative disorder of the joints and the leading cause of pain and disability globally. OA has been reported to affect more than 303 million people worldwide in 2017, and the socioeconomic cost related to it is increasing rapidly [25]. OA is also implicated in increasing the risk of cardiovascular-related mortality [26] and suicidal thoughts [27]. The prevalence of OA is expected to rise drastically over the years, concomitant with the increase of the aged and obese population [28].

**Figure 1:**
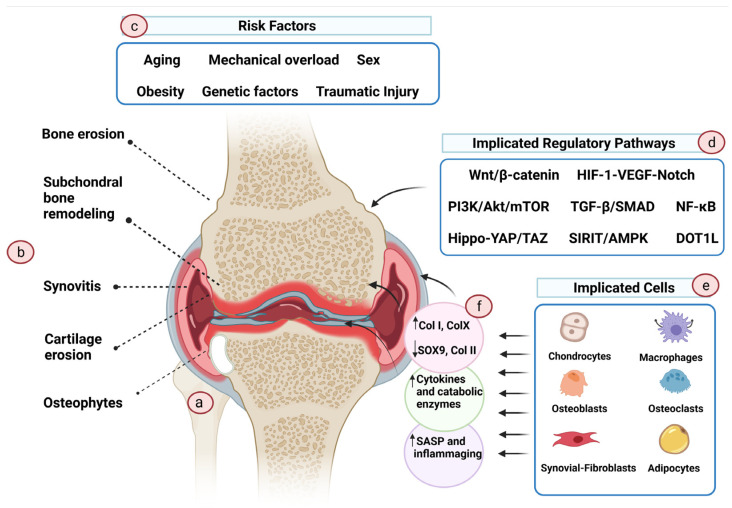
Pathogenesis of osteoarthritis. OA is a complex disease with a wide range of risk factors and causes. (**a**) OA knee. (**b**) OA affects not only the cartilage tissue but also the surrounding tissues, causing modulation of the subchondral plate, inflammation of the synovial tissue, and erosion and osteophyte formation at the supporting bone. (**c**) The primary causative event of OA is largely unknown. Aging, chronic mechanical overload, traumatic injury, genetic variables, sex, and associated hormonal variation are some of the risk factors that have been proposed to increase the vulnerability to OA. (**d**) Multiple regulatory pathways are involved in OA onset and progression, but not all are necessarily implicated in all phenotypes of the disease. (**e**) Cells in the affected tissues actively participate in the disease’s initiation and propagation. (**f**) During OA, these cells change phenotype and, as a result, exhibit altered transcriptome profiles that promote an endless cycle of inflammation and tissue deterioration. (Created with BioRender.com on 11 August 2021).

OA is often described as a heterogeneous, multifaceted “syndrome” with varied pathogenic pathways, phenotypes, clinical presentations, and prognosis; however, these heterogeneous forms of OA have a similar endpoint, i.e., joint destruction, leading to functional disability [29,30]. A recent study attempted to decipher the genetic variants that might contribute to the genetic predisposition to OA. Surprisingly, no overlap was found in the genetic variants that predispose to hip and knee OA, highlighting the vast heterogeneity of this disease [31]. OA is characterized by the progressive remodeling of joint tissues, propelled by a multitude of inflammatory factors within the joint [32]. OA does not only irreversibly affect the hyaline cartilage, but, rather, the damage also extends to distort all other components of the joint, including the subchondral bone, meniscus, ligaments, and synovial tissue (Figure 1) [32].

Although the initiating event that causes OA development is still unknown, results from preclinical and clinical studies suggest that older age, obesity, genetic factors, traumatic injuries, chronic mechanical overload, sex (female) and hormone levels, and metabolic disorders are the risk factors for OA [33]. Several cell types have been recognized to aggravate the OA pathological process. These include osteoblasts of the subchondral bone [34], M1 macrophages [35] and fibroblasts of the synovial tissue [36], adipocytes of the infrapatellar pad [37], and senescent and hypertrophic chondrocytes [38,39]. In OA, these cells are dysfunctional, exhibiting aberrant gene expression profiles and phenotypes. Conversely, in healthy cartilage, chondrocytes are quiescent, resuming stable phenotype and resisting “hypertrophic differentiation”—the process that is important for bone growth by endochondral ossification of the epiphyseal growth plate in children and adolescents [40]. OA chondrocytes appear to escape the maturational arrest and start to acquire the hypertrophic phenotype, whereby the cells demonstrate altered morphology, increase in size, and form clusters [39,41]. Additionally, hypertrophic OA chondrocytes overproduce non-cartilage matrix constituents such as type I collagen and type X collagen, cartilage-degrading enzymes such as matrix metalloproteinases (especially MMP13), and A Disintegrin and Metalloproteinase with Thrombospondin motifs (ADAMTS), as well as proinflammatory cytokines and chemokines [42,43]. Uncertainty still shrouds our understanding of the fate of hypertrophic chondrocytes, whether they end up differentiating into bone cells or undergoing apoptosis [39]. Nonetheless, apoptosis has been implicated in lacunar emptying and hypocellularity within OA cartilage, which is noticeable in advanced OA [44,45]. Chondro-senescence has also been suggested to contribute to the pathogenesis of OA. The proportion of senescent cells in joints is tightly linked to age [46,47]. Senescent cells adopt a distinct senescence-associated secretory phenotype (SASP). SASP is characterized by the overproduction of proteolytic and proinflammatory factors and the generation of reactive oxygen species (ROS), which are deleterious to the surrounding tissue, consequently inducing joint damage [48]. Premature chondrocyte senescence can be induced by injury [49,50], mechanical stress [49], and obesity, leading to early-onset OA [48].

Several signaling pathways have been implicated in OA development and progression. These pathways include Wnt/β-catenin, PI3K/Akt/mTOR, DOT1L, SIRIT/AMPK, Hippo-YAP/TAZ, NF-κB, NLRP3 inflammasome-mediated pyroptosis, and HIF-1-VEGF-Notch pathways [33,51,52,53]. Another important pathway involved in the inflammatory progression of OA is the mitogen-activated protein kinases (MAPK) pathway, which comprises the extracellular signal-regulated kinase 1/2 (ERK1/2), the c-Jun N-terminal kinase (JNK), p38, and ERK5 cascades [54,55]. In addition, a number of miRNAs and lncRNAs have also been identified to mediate OA pathogenesis [33]. The unraveled pathways serve as potential targets for OA therapies. However, OA subtypes exhibit variable pathophysiological pathways, rendering the development of effective therapies challenging.

## 3. Management of Osteoarthritis

Effective treatment for OA is still an unmet need. Present OA management regimens focus on multiple approaches such as patient self-monitoring and symptom modification [56], while disease-modifying treatment options are still lacking [57]. For the self-monitoring option, patients are educated on the course of the disease, assisted in reducing weight, increasing exercise, and undergoing physiotherapy [56]. Pharmacological options are mainly for symptom and pain relief. Drugs such as paracetamol, nonsteroidal anti-inflammatory drugs (NSAIDs), COX inhibitors, intra-articular corticosteroid injections, and opioids are commonly prescribed, although Osteoarthritis Research Society International (OARSI) guidelines have warned against excessive use of these drugs due to the risk of severe side effects [58].

Currently, there is no disease-modifying osteoarthritis drug (DMOAD) that has been approved by the regulatory communities, even though a number of candidates have been or are being tested in phase II and III trials [59]. Promising results have been reported in some of the clinical trials. DMOADs aim to modify the underlying pathological course of the disease by targeting cartilage metabolism, subchondral bone remodeling, and/or inflammation [59,60]. Considering the absence of disease-modifying therapeutics, OA treatment relies on invasive total joint replacement in advanced OA. Early surgical attempts by techniques such as Pridie drilling, abrasion arthroplasty, and Steadman’s microfracture have aimed at disrupting the vasculature and marrow through the mechanical penetration of the subchondral bone to permit the efflux of marrow elements to the defective site [61,62], leading to a natural inflammatory response and successive recruitment of the mesenchymal progenitor cells to promote cartilage regeneration [61,62]. Although these interventions eventually result in the regeneration of fibrocartilage tissue of suboptimal properties, they also have the potential of stimulating the intrinsic repair process to restore native cartilage tissue. Of note, these surgical interventions are only applicable to younger patients (<40-year-old) with smaller defects (<2 cm^2^), as older patients may suffer from limited regenerative capabilities for cartilage restoration [63].

Cell-based regenerative approaches have been introduced for cartilage repair since 1994 [64]. Thus far, cell therapies, especially autologous chondrocyte implantation (ACI) and mesenchymal stem cell (MSC) transplantation, have shown some success in preclinical and clinical studies and received market authorization in several countries. However, ACI success was only shown in young patients with localized small lesions (>2.5 cm), but not in older patients with degenerative cartilage or larger defects [65,66]. Other limitations such as painful cartilage sample collection, dedifferentiation of cultured chondrocytes, and long expansion period to obtain sufficient cells for transplantation render ACI less popular compared to MSC therapy [3]. In addition, allogeneic MSCs are mildly immunogenic, anti-inflammatory, and can be expanded on large-scale relatively easily [7,67,68]. MSCs are known to differentiate into chondrocytes and secrete paracrine factors to facilitate cartilage regeneration [69,70]. The therapeutic benefits of MSC therapy for OA management have been reported in several clinical studies [71]. Although stem cell therapy has been shown to be safe, with no serious adverse effects, there are still some concerns over the treatment’s sustainability, standardization, biosafety, and operational and logistical feasibility throughout cell production and transportation [71,72]. Experiments tracking delivered stem cells have consistently revealed low cell engraftment below 3% in the treated joints [73]. Therefore, the improvement in the treated joints is considered to be mainly attributed to the cells’ paracrine secretion. MSCs release a battery of biomolecular cues that modulate the host microenvironment, promoting host tissue repair mechanisms as well as immunomodulating and disrupting the OA pathological cascades [73]. EVs, especially exosomes, have been suggested to be a vital mediator of MSCs’ paracrine effect and serve as a promising cell-free therapeutic candidate for OA [72].

Recent years have witnessed the emergence of new promising approaches in OA management, such as machine learning (ML). This comprehensive computational approach is very promising and is gaining increased interest. ML is a powerful tool with algorithms that can analyze large datasets, integrate diverse data, and analyze patterns that, collectively, could help in the endeavor to categorize OA phenotypes. ML could greatly assist in unraveling the biological mechanisms underlying OA development, predicting responsiveness to treatment, and simulating the processes in disease progression [74]. This revolutionary approach would allow data-guided decision-making and support personalized treatment options [75]. Despite its existing shortcomings, which may compromise its credibility, ML is continuously evolving, and promising advances have been made at a very fast pace. As a result, we anticipate that this approach will aid in a more precise diagnosis of OA and the selection of the best treatment options.

In light of the discussed issues pertaining to OA development and management, in the following sections, we endeavor to provide an overview of the relatively new approach of utilizing secreted small EVs as a biological cell-free treatment in cartilage regeneration as well as their unique miRNA content and their contribution to advancing the field of regenerative medicine.

## 4. Exosomes

### 4.1. Categories

Exosomes and ectosomes, which are derived from endosomes and the plasma membrane, respectively, are the two subtypes of lipid-bound EVs secreted into the extracellular environment. These EVs do not possess a nucleus structure; hence, they cannot replicate. According to the latest position statement indicated in the Minimal Information for Studies of Extracellular Vesicles 2018 (MISEV2018) report, exosome and ectosome terminologies should only be used if an experimental research design can establish the subcellular origin of the EV subtype being studied. Otherwise, operational terminologies for the subtypes of EVs should be adopted, which are based on (i) particle size (small EVs for particles smaller than 200 nm, and medium and large EVs for particles larger than 200 nm), (ii) surface markers expression or biochemical recognition (such as CD81+, CD63+ or annexin A5-stained), and (iii) the origin or condition of cells where the EVs are isolated (such as hypoxic EVs, apoptotic bodies, and podocyte EVs) [76].

Exosomes can be either native or bioengineered, depending on whether they have been artificially manipulated or not. Both animals and plants can produce native exosomes, hence giving rise to animal-based exosomes or plant-based exosomes. Among the animal-based exosomes, they can be further classified depending on the type of cells that are producing the exosomes—either exosomes produced by normal and healthy cells or those that are synthesized from tumor cells. Amongst the variety of normal cells that can produce exosomes, MSC-derived exosomes have been given considerable attention due to the capability of MSCs to self-renew and undergo multilineage differentiation. Therefore, MSC-derived exosomes are being actively investigated for their tissue regeneration potential [77].

### 4.2. Biogenesis

Exosomes, the smallest form of EVs, with diameters ranging from 30–150 nm [78], are usually synthesized through an endosomal-sorting complex required for transport (ESCRT)-dependent or ESCRT-independent pathways [79,80,81,82]. The ESCRT-dependent pathway for exosome biogenesis usually involves a series of budding processes that are facilitated by complex protein machinery, which is primarily made up of four distinct ESCRT proteins (0 to III). This pathway is triggered when ubiquitinated proteins are recognized by ESCRT-0 and are sequestered into specific endosomal membrane domains. After further interacting with ESCRTs I/II and forming a total complex with ESCRT III, plasma membrane infolding begins, resulting in the formation of a cup-shaped early endosome that contains cell-surface proteins and soluble bioactive substances that are accumulated from the extracellular environment. As this early endosome buds inward, it matures into a late endosome that subsequently undergoes a secondary endosomal membrane invagination to form a multivesicular body containing intraluminal vesicles [80,83,84,85,86]. These intraluminal vesicles have similar membrane positioning to the cell surface, where extracellular domains of transmembrane protein face the extracellular environment while enclosing the cytosolic entities [87]. As the multivesicular body moves to the cell surface and fuses with it, the intraluminal vesicles are released into the extracellular space, giving rise to exosomes [77]. Other than ESCRT proteins, numerous other accessory proteins have been shown to be involved in the production of exosomes through the ESCRT-dependent pathway, including programmed cell death 6-interacting protein (ALIX); tumor susceptibility gene 101 protein (TSG101); heat shock cognate protein 70 (HSC70); vacuolar protein sorting-associated protein 4 (VPS4); heat shock protein 90 (HSP90); soluble N-ethylmaleimide-sensitive fusion attachment protein receptor (SNAREs); cluster of differentiation proteins 9, 81 and 63; and syndecan-1, synthenin-1, and tetraspanins [79,85,87,88]. In the alternative ESCRT-independent pathway, ceramides play an important role as their cone-shaped feature is speculated to promote microdomain-induced budding of the endosomal membrane. These ceramides are usually produced through the removal of the phosphocholine moiety by sphingomyelinases that are abundantly present in those microdomains. The presence of ceramides will also induce the lateral isolation of cargo within the endosomal membrane that leads to the formation of intraluminal vesicles within the multivesicular body, releasing exosomes once it fuses with the plasma membrane [89,90].

### 4.3. Therapeutic Cargos of Exosomes

Upon secretion into the extracellular space, exosomes will migrate and deliver their contents to their target cells, resulting in the alteration of gene expression as well as the modification of physiological and biological functions. The functional modification relies on the type of exosomal contents present within the exosomes [91]. Invariably, most exosomes are rich in proteins and lipids. To date, about 8000 proteins and 194 lipids have been found to be related to exosomes [92]. Along with these, exosomes are known to carry nucleic acid cargos, including mRNAs and miRNAs, for intercellular communication [93]. Since exosomes are secreted by numerous cell types and the exosomal content is heavily associated with the cell of origin, each exosome may have different roles in intercellular communication for varying physiological effects. For example, platelets secrete exosomes containing prostaglandins that modulate inflammatory activities [94]. Meanwhile, antigen-presenting cells, such as dendritic cells, release exosomes that carry functional major histocompatibility complexes class I and II (MHC I and MHC II) to attenuate or stimulate antigen-specific B-cell or T-cell responses [95]. Similarly, many other studies have also demonstrated the involvement of exosomes in various biological processes, including coagulation, blood vessel formation, red blood cell maturation, and removal of unwanted RNAs and proteins [96].

In view of exosomes’ capability to carry their cargo to the target cells for participation in both normal and pathobiological mechanisms through intercellular communication, extensive investigations have been performed to exploit their therapeutic purposes to restore the diseased tissue to its normal phenotype. One of the approaches involves the isolation of these endogenous exosomes from body fluids so that they can be artificially enriched with therapeutic miRNAs before these bioengineered exosomes are reintroduced back into the patient’s body [93,96]. Exosomes have increasing popularity as a preferred drug delivery vehicle as they are non-immunogenic. In addition to that, other advantages of exosomes over MSCs are their homing ability to the site of injury and passing through tight junctions such as the blood–brain barrier. Since exosomes are very heterogeneous, they can also carry different proteins on their surface that can be delivered into the cell through receptor-mediated endocytosis upon interacting with the target cells [77]. Therefore, the exosome-assisted delivery approach has been utilized by various research groups to deliver a variety of therapeutic compounds to their target cells, including short interfering-RNA (siRNA), recombinant proteins, miRNA, and antagomirs, as well as anti-inflammatory and chemotherapeutic drugs [89]. For instance, exosomes equipped with siRNAs against the mRNA of Huntington’s disease have been successfully introduced into the cortical neurons of mice, resulting in an improvement of the murine Huntington’s disease condition [97]. Through the use of exogenous siRNAs, the MAPK gene in target lymphocytes and monocytes could be silenced. Hence, this proves the feasibility of bio-engineering isolated exosomes for the targeted delivery of therapeutic materials to a specific tissue to exert its function [98]. Alternatively, donor cells can be bio-engineered to contain the therapeutic materials, which will then be synthesized and encapsulated in the exosomes produced. These enriched exosomes can then be used for the treatment of specific diseases. An example would be a study done by Shimbo et al. that introduced miR-143 into MSCs, where this miRNA was then secreted and packaged into the exosomes. Upon delivery to osteosarcoma cells, the miR-143 present within the exosomes were able to significantly inhibit the migration of the cancerous cells [99]. In view of these and many other successful examples in utilizing exosomes as a delivery vehicle for therapeutic purposes, exosomes have been accepted as drug carriers for the treatment of various diseases in clinical trials [79].

## 5. Promoting Cartilage Repair Using Exosomes

Plastic and reconstructive surgery can be used to restore the structure and function of cartilage defects. However, these treatments are more successful for minor defects. When it concerns a sizable cartilage defect, such treatment options still pose some limitations. Therefore, autologous or matrix-assisted implantation of chondrocytes could be a more effective treatment. Unfortunately, the use of these therapeutic approaches is also restricted by the lack of donor sites as a result of possible post-donation adverse effects as well as risks associated with the deterioration of graft tissue [100]. Recently, researchers have resorted to utilizing exosomes derived from stem cells as an alternative therapy due to the ease of accessibility, unlimited supply, and better stability. Besides that, exosomes are secretory products enriched with active molecules that could result in the desired therapeutic outcomes [101]. The following paragraphs describe the composition of exosomes that are desirable for therapeutics.

Firstly, exosomes contain various types of growth factors and microRNAs that can stimulate cartilage regeneration. This notion arose through a study that demonstrated the successful repair of injured cartilage tissue when it was co-cultured with MSCs. It was eventually discovered that the cartilage tissue regeneration was achieved due to the presence of different growth factors secreted by MSCs, including fibroblast growth factor (FGF) 2, interleukin (IL)-6, and insulin-like growth factor (IGF). These biological factors possess the capability to induce the proliferation and matrix synthesis of chondrocytes, thus assisting in the healing of the injured cartilage [102]. In agreement with this, Jiang et al. observed enhanced osteochondral regenerative activity in the presence of exosomes derived from human umbilical cords. These exosomes were able to induce the proliferation and migration of chondrocytes, hence repairing the osteochondral knee injury in a rabbit model. The regenerative potential of the exosomes in this study was attributed to the presence of about 20 miRNA types that may exert positive effects in the regulation of the joint microenvironment [103]. Similar findings were observed in another study in which complete recovery of osteochondral defects in a rat model was achieved after treatment with MSC-derived exosomes. The restored cartilage and osteochondral bone displayed normal structural characteristics that resembled their normal counterparts, such as hyalinized cartilage with ideal surface regularity and good attachment to the adjoining cartilage, with well deposited ECM [104].

Other than their regenerative capacity, exosomes were also found to possess chondroprotective effects. For instance, treatment of synovial explants with MSC-conditioned media was found to inhibit the expression of matrix degradative enzymes, including matrix metalloproteinase (MMP)-1, MMP-12, and IL-1b, thus facilitating the repair of cartilage tissue [105]. A separate in vitro study also pointed towards the aptitude of MSCs to protect chondrocytes in cartilage via the upregulation of type II collagen production [106] to resynthesize the matrix. Apart from restoring the cartilage matrix, enhanced expression of type II collagen could also prevent the hypertrophy of chondrocytes, thus avoiding the progression of cartilage degeneration [107]. Moreover, this chondrogenic protective effect has been demonstrated by Cosenza et al., who showed the ability of MSC-derived exosomes to exert protective effects on chondrocytes in a murine model with induced joint disease. This chondroprotective outcome was achieved through an increased expression of chondrocyte markers (such as aggrecan and type II collagen) while suppressing catabolic genes (including ADAMTS5 and MMP-13) to prolong the survival of OA-like chondrocytes induced with IL-1β [108]. Additionally, MSC-derived exosomes potentially assisted in cartilage repair by increasing the number of chondrocytes by preventing chondrocyte apoptosis through the upregulation of anti-apoptotic proteins, including Bcl-2 and survivin [109].

Post-traumatic inflammation almost always occurs alongside cartilage injury, and it is a huge obstacle for cartilage repair. This is because cartilage is an avascular tissue; hence, they do not have the capability to resolve an inflammatory response, resulting in the cartilage being easily assaulted by the proinflammatory mediators [110]. Thus, it becomes a critical therapeutic obstacle to suppress inflammation prior to the repair of injured cartilage via regeneration. In this aspect, MSC-derived exosomes have proven to be valuable as they can exert an anti-inflammatory response. For example, adipose MSC-derived exosomes could decrease levels of pro-inflammatory mediators, reducing stimulation of NF-κB and activator protein 1 while increasing expression of anti-inflammatory cytokines and the expression of miR-100-5p, which binds to 3′UTR of mTOR to augment autophagy activity [8,111]. With similar mechanisms, exosomes isolated from bone marrow-derived MSCs [112] and umbilical-cord-derived MSCs [113] were able to relieve the pathological characteristics in diseased joints through decreasing the infiltration of inflammatory cells as well as repressing levels of inflammatory factors [114].

## 6. Exosomal miRNAs

The cargo of stem cell-derived exosomes has been widely studied since 2010 when the therapeutic potential of MSC-derived exosomes was first described [115]. Although exosomes are enriched in many bioactive molecules, such as lipids, proteins, RNA, and mtDNA [116], the therapeutic potential of MSC-derived exosomes is usually rationalized with the presence of biologically relevant miRNA and proteins [117]. To date, more than 1000 proteins have been identified and mapped in MSC-derived exosomes, suggesting that the proteome of MSC-derived exosomes play a vital role in various biological processes such as cellular communication, exosome biogenesis, and tissue repair [118,119,120]. Similar to the MSC-derived proteins, many studies have suggested that MSC-derived miRNAs have the potential to modulate cell–cell communication as well as influence the progression of various diseases by regulating the signaling pathways of the recipient cells. In this paper, we focus on exosomal miRNAs that are biologically relevant to OA.

MSC-derived exosomes carry many RNAs, including messenger RNA (mRNA), long non-coding RNA (lncRNA), small non-coding RNA (microRNA, small nuclear RNA, and Piwi-interacting RNA), Y-RNA, ribosomal RNA (rRNA), and transfer RNA (tRNA) [121]. Interestingly, results from both microarray and next-generation sequencing studies suggested that the packing and secretion mechanisms of exosomes are not random. Selective processes such as 18S or 28S RNAs or RNAs larger than 500 nucleotides were not detected in MSC-derived exosomes [122]. One way in which exosomes can modulate the target cell function was suggested to be through the transfer of enclosed mRNA. It has been shown that mRNAs in exosomes are translatable, which leads to the production of specific proteins. However, the physiological significance of such mRNAs for cellular functions remains unclear as mRNAs only contributed to a small proportion of the RNAs enclosed within the exosomes [123,124].

The miRNAs are a group of small, non-coding single-stranded RNAs, averaging 19–24 nucleotides, that regulate post-transcriptional gene expression. miRNAs are essential for physiological development and are involved in a variety of biological processes [125]. Abnormal expression of miRNAs is associated with several human diseases [126]. In addition to intrinsic cellular functions, miRNAs are secreted into extracellular fluids via EVs as signaling molecules mediating intercellular communication [127,128,129,130]. Hypothetically, each functional miRNA can interact with up to 200 mRNAs [131]. Intercellular communication can occur by several means, including receptor-mediated chemical interaction, direct cell–cell communication, and cytosolic synapses. These intercellular gene communications may occur not only in the microenvironment but can also occur at a distance through the secretion of exosomes into systemic circulation. Indeed, exosomes may be a more effective intercellular communication option, compared to proteins or small biochemical molecules such as mRNAs and miRNAs, that can regulate recipient cell protein production and gene expression. The ability of exosomes to deliver genetic material into cells at a distance also makes them ideal candidates for cell-free therapy.

### 6.1. Biogenesis of miRNA

**Figure 2:**
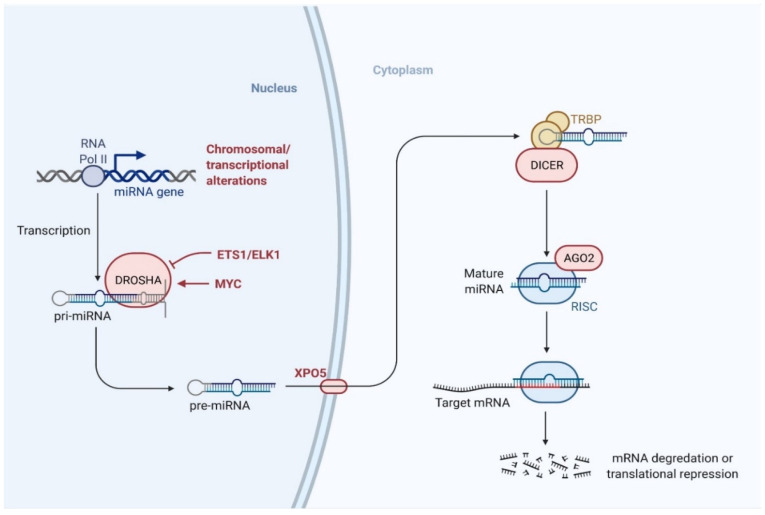
Biogenesis pathway of miRNA. miRNAs are processed by two successive steps in humans: first, in the nucleus by Drosha, followed by Dicer in the cytoplasm. The mature miRNA then binds to the argonaute (AGO) family of proteins to form the core of the RNA-induced silencing complex (RISC) and interacts with the target mRNAs to regulate gene expression. (Created with BioRender.com on 23 July 2021).

miRNAs consist of approximately 19–24 nucleotides and originate from precursors with a characteristic hairpin-shaped loop structure [132]. miRNAs have a phosphorylated 5′-end and a 3′-end with two hydroxyl groups and are normally processed by two successive steps in humans: first, in the nucleus by an RNase III called Drosha, followed by a second RNase III called Dicer in the cytoplasm (Figure 2). These enzymes cut the genome-encoded stem-loop precursors into the mature miRNA [133]. The mature miRNA attaches to an argonaute (AGO) protein to form the core of the RNA-induced silencing complex (RISC), which subsequently interacts with target mRNAs to regulate gene expression.

### 6.2. Mechanism of miRNA-Mediated Gene Regulation

According to previous studies, miRNA-mediated gene regulation is dynamic. miRNAs bind to specific regions in the 3′ UTR of target mRNAs to cause translational repression, mRNA arrest, and unwinding in general [134,135]. However, miRNA can also bind to other mRNA regions, including the 5′ UTR and coding sequences as well as within promoter regions [136]. miRNAs can regulate the gene expression via multiple pathways by forming RNA effector complexes, such as miRgonaute, miRNP, or miRISC [137,138]. The key factor for miRNA target recognition depends on the pairing of the Watson and Crick sequence to the proximal 5′ “activating” region (located at nucleotide 2–8) of the miRNA to the corresponding site in the target mRNA at 3′ UTR [139]. However, studies have also suggested that certain miRNAs may influence expression by selectively targeting the 5′ UTR and/or coding area of particular mRNAs [136]. In addition to the binding position, other factors such as binding and repression strength, number of target sites, RNA secondary structure, site accessibility, and sequences flanking may also influence the gene regulation potential [140,141,142]. Over the decades, many studies have been conducted to study the potential of MSC-derived exosomes for treating OA and to identify the miRNAs that play a vital role in maintaining a healthy joint (Table 1).

## 7. Modulating the miRNA Content of Exosomes

**Figure 3:**
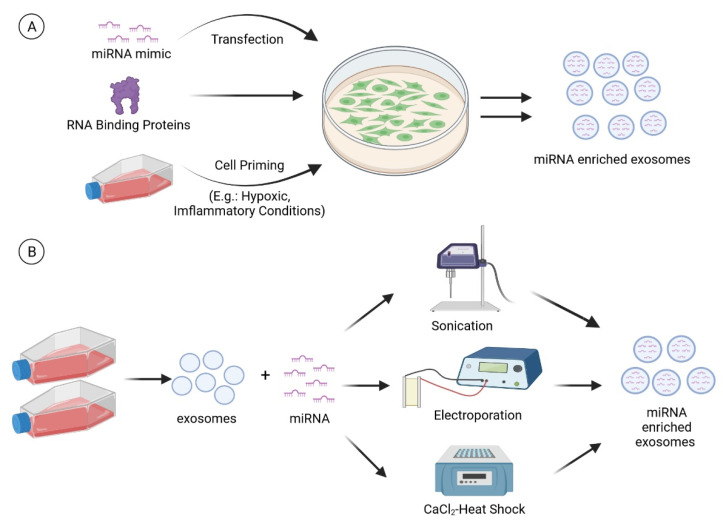
miRNA enrichment can be performed via (**A**) cell modification or (**B**) direct loading of miRNAs into exosomes. For cell modification, a miRNA mimic is transfected into the cells. In addition, RNA binding proteins and cell priming (e.g., hypoxia and inflammatory cytokines) strategies can also be employed to modulate the miRNA cargo of exosomes. Techniques such as sonication, electroporation, and CaCl_2_-heat shock can be used to load the miRNAs directly into the exosomes. (Created with BioRender.com on 28 July 2021).

miRNAs encapsulated and transferred by MSC-derived exosomes have been documented as an important therapeutic factor to enhance chondrogenesis and suppress cartilage degradation. With these exciting findings, attempts have been carried out to enrich specific therapeutic miRNAs in EVs for a more predictable and desirable clinical response [151,153,154]. In general, miRNA enrichment could be performed via the cell line overexpressing technique or via direct loading of miRNAs into exosomes using physical or chemical methods (Figure 3).

### 7.1. Cell Modification

#### 7.1.1. Transfection

Mammalian cell transfection techniques have been widely used and established in the past decades to overexpress specific therapeutic factors such as miRNAs and proteins to investigate their mechanism of action in the recipient cells or disease models. These discoveries have been translated into clinical applications. An example is the transfection of miRNA mimics into MSCs to obtain exosomes with enhanced therapeutic potential for the treatment of cartilage diseases. The selection of transfection protocol (viral, hybrid, and non-viral approaches through either physical or chemical methods) is critical to ensure high transfection efficiency in MSCs. The various transfection protocols have been discussed in detail in previous papers [159,160]. The transfected cells may transiently or permanently express the exogenous miRNAs and pass them to the next generation. Thus, it is important to monitor the concentrations of target miRNAs in the exosomes as this is a crucial factor that influences therapeutic efficiency. In addition, it is also advisable to monitor the secretion of exosomes by the transfected cells and determine the uptake of the bioengineered exosomes by the target cells. To date, various sources of MSCs have been employed to overexpress specific miRNAs such as miR-140-5p [154], miR-92a-3p [151], miR-100-5p [153], and miR-155-5p [161] to produce miRNA-enriched exosomes for the treatment of OA.

MSC is a popular cell of choice for transfection due to the convenience of isolation and large-scale expansion in the laboratory. In particular, Wharton’s jelly-derived MSCs (WJ-MSCs) can be harvested from the umbilical cord with ease and non-invasively. “Young” WJ-MSCs are preferred over the autologous MSCs from aged OA patients due to the poor growth of the collected cells from aged OA patients, which results in a lower yield of exosomes. As an alternative, researchers have also transfected the immortalized MSCs derived from induced pluripotent stem cells (iPSCs) and embryonic stem cells (ESCs) to produce exosomes for therapeutic applications [162,163]. Nevertheless, the use of exosomes secreted by the immortalized MSCs is hindered by the concern of the transfer of pro-oncogenic materials that may cause tumorigenesis.

#### 7.1.2. RNA Binding Proteins

RNA binding proteins (RBPs) play an important role in sorting and packaging miRNA into EVs, suggesting that RBPs could be enriched or silenced in stem cells to modulate the miRNA content in EVs. Statello et al. (2018) reported that a total of 9 RBPs (MVP, PCBP1, HSPA8, MOCS3, SNW1, EEF1A1, HNRNPK, HnRNPM, and HSP90AB1) isolated from exosomes formed miRNA-RBP complexes [164]. To further identify the exact RBPs that play a role in the transport of miRNA into exosomes, gene transcripts encoding MVP, HNRNPM, and HSP90AB1 were silenced in cells using short interfering RNAs. These three exosomal-RBPs were selected because they are involved in different stages of miRNA metabolism, splicing, maturation, and RNA transport. Results showed that the silencing of HNRNPM and HSP90AB1 did not reduce the amount of total RNA present in the exosomes. However, the silencing of MVP caused a 50% reduction in total RNA present in the exosomes, indicating its critical role in miRNA transport into exosomes.

In other studies, Villarroya-Beltri and colleagues demonstrated that hnRNPA2B1 recognized the GGAG/UGCA motif in miR-198 and miR-601. The silencing of hnRNPA2B1 decreased the miRNA in exosomes by 13%, suggesting its role in the exosomal sorting of miR-198 and miR-601 [165]. Another RBP, SYNCRIP, was also reported to recognize miR-3470 and miR-194-2-3p. The silencing of SYNCRIP decreased the concentration of miR-3470 [166]. Nevertheless, it should be noted that different cell types may employ RBPs with distinct binding preferences or multiple RBPs to secrete and sort miRNAs into exosomes [167]. Sometimes, more than one RBP is involved in miRNA sorting. For instance, caveolin-1 assists in the sorting of miRNAs, along with hnRNPA2B1 [168].

The above-mentioned studies on RBPs were discussed in relation to other cell types not related to stem cells. Although RBPs have been identified in ESCs, nevertheless, to the best of our knowledge, no study has attempted to definitively identify the RBPs that are responsible for transporting miRNA into stem-cell-derived exosomes. Despite the gaps in knowledge about the function of RBPs in stem cells, previous studies have shed some light on the possible RBPs that may be responsible for such a role in stem cells, and this warrants further exploration. This will pave the way for the utilization of RBPs in developing and improving miRNA-enriched EV therapies.

#### 7.1.3. Cell Priming

Cell priming has long been used as a strategy to enhance the survival and biological activities of stem cells for the treatment of diseases. For instance, the hypoxic condition has been reported to enhance chondrogenesis and the production of functional cartilage from stem cells [169,170,171]. Similarly, inflammatory priming with TNFα, IL-1β, or interferon-gamma (IFNγ) increases the immunomodulatory properties of MSCs in vitro and in vivo [172,173,174]. Although studies have reported that the culturing of stem cells under hypoxic and inflammatory conditions modified the secretome [174,175,176] and EV profiles [177], nevertheless, it seems the priming has minimum effects on the miRNA landscape of MSC-derived EVs [178]. Peltzer et al. reported that only five miRNAs (hsa-miR-25-3p, hsa-miR-106a-5p, hsa-miR-126-3p, hsa-miR-451a, and hsa-miR-665) were upregulated in response to IFNγ priming, whereas hypoxia priming downregulated only hsa-miR-34a-5p. The finding was also in line with another study in which IFN priming merely affected four miRNAs in endometrial MSC-derived EVs [179]. Interestingly, the overexpression of hsa-miR-25-3p [180], hsa-miR-106a-5p [181], hsa-miR-126-3p [182], and hsa-miR-665 [183] has recently been reported to inhibit chondrocyte apoptosis and/or suppress chondrocyte inflammation and cartilage degradation. Another study uncovered the role of hsa-miR-34a-5p, which promotes joint destruction during OA [184]. Based on the above information and direct interpretation, it seems priming could enrich cartilage-protecting miRNAs and downregulate cartilage-degrading miRNAs in exosomes. Although the information may be valid, the modification to the miRNA profile in EVs is random and not targeted.

### 7.2. Direct Introduction of miRNA into Extracellular Vesicles

Apart from cell transfection, direct introduction of the desired miRNA into EVs is also a highly applicable and amenable approach to enrich miRNAs in the EVs’ cargo. This can be achieved by incubating EVs with the desired miRNAs, with or without a calcium chloride (CaCl_2_) buffer [185,186]. It has been reported that CaCl_2_ enhances miRNA uptake into the EVs since CaCl_2_ can promote the interactions between miRNAs and the EV surface [186]. In addition, heat shock can also be used to change the fluidity of the exosomal membrane, which facilitates the entry of miRNA [186]. Apart from CaCl_2_–heat shock, electroporation is another technique that can be used [185,186,187]. This process uses voltage and pulse to open the EVs’ pores to facilitate miRNA entry. Although the idea sounds great, nevertheless, the use of electroporation for EVs is still in the infancy stage, and the existing limitations would require further attention. First, the optimization of the voltage and pulse for electroporation is critical since the opening of the EVs’ pores is cell-dependent due to different membrane protein compositions [185]. Second, contamination, such as from the probe and buffers, is also a concern. Third, the aggregation of EVs and miRNA may impact the functionality of the nucleic acids [187]. Fourth, the leakage of endogenous cargo, including miRNA and proteins during electroporation, may alter the therapeutic properties of the EVs. Some of the limitations have been explored and resolved by Pomatto et al. and colleagues; nevertheless, improvements are still needed to further refine the technique for future applications [185,186].

## 8. Delivery of Exosomes for Cartilage Repair

Drug delivery technology has come a long way since it first started in the 1950s [188]. During the first generation of delivery advancement, extensive effort was placed on optimizing oral and controlled release formulation. The major challenge was to overcome the physicochemical properties of active pharmaceutical ingredients (APIs). Thirty years later, when oral delivery technology had become mature, researchers advanced to the second generation of delivery technology. This time, the physicochemical problems posed by APIs no longer significantly impact formulation scientists, while, on the other hand, overcoming the physiological barrier is of prime importance. Most of the delivery technologies have focused on drugs or compounds that had been marketed, mainly small molecules. While we were transitioning towards the third generation of delivery technology, EVs appeared. The discovery of EVs such as exosomes has completely shifted the paradigm of traditional drug delivery technology. Being sphere-like vehicles, EVs can entrap a variety of compounds or APIs that would otherwise have had to be delivered one by one using traditional drug delivery designs. The delivery of exosomes is, therefore, very different from small molecules.

The efficient delivery of exosomes, including the mode of administration, is governed by the physicochemical properties of the membrane bilayer instead of the cargo within the exosomes. Unfavorable conditions such as extreme pH, osmolarity, mechanical stress, the presence of surfactants, and certain enzymes notably affect membrane integrity and can lead to the destabilization of the membrane bilayer. Consequently, the premature exposure of cargo materials to the environment before reaching the target site can lead to their degradation. In view of these stringent storage environments, the preservation of exosomes over an extended period, before and during administration, is challenging. Additionally, conventional easy-to-apply delivery methods such as transdermal, oral, buccal, and sublingual seemed to be unattainable currently. Research using exosomes for cartilage regeneration is generally administered via needle injection.

Biologic therapies for regenerative medicine have opened a huge window for a plethora of clinical applications. As the area advanced from bench to bedside, the surge of interest in exosomes also induced the emergence of compliance guidelines. The approval to use exosomes for therapeutic purposes is required to comply with the US Food and Drug Administration (FDA) regulation of human cells, tissues, and cellular and tissue-based products (HCT/Ps) [189]. The compliance guidelines are regulated under Title 21, Part 1271 of the Code of Federal Regulations (CFRs). The document has specifically highlighted several aspects that emphasize, i.e., HCT/Ps are minimally manipulated, for homologous use, have no systemic effects, and are not used in combination with and are not dependent on the metabolic activity of living cells. Regenerative medicine researchers should be wary of the implementation of the new regulations because the criteria set forth might impact the trajectory of experimental planning. Some of the changes are significant and could potentially affect product registration.

### 8.1. Direct Needle Injection: Exosome Gel and Formulation

Direct needle injection to the target area represents the most preferred method of administration pertaining to cartilage repair. This can be seen in many published clinical trials. One of the recently published trials examined the safety and efficacy of umbilical-cord-derived Wharton’s jelly in human knee OA [190]. The injectable formulation was applied with minimal alteration to adult patients over the age of 18 years diagnosed with grade II or grade III (mild or moderate) OA. However, it is noteworthy that the exosomes were a part of the complex pool of extract derived from Wharton’s jelly [191]. Apart from the functional properties that the extract may have, the jelly also provides support that retains exosomes within the injectable site for a longer period.

Tao and colleagues also adopted direct injection via the intra-articular route in Sprague–Dawley rats [192]. The rat OA model was established via surgical destabilization of the medial meniscus. The exosomes were processed and isolated from synovium MSCs. Instead of an injection, the exosomes were dispersed in liquid, and the team mixed the exosomes with a type of polymeric gel, i.e., poly(D,L-lactide)-b-poly(ethylene glycol)-b-poly(D,L-lactide) (PDLLA-PEG-PDLLA, PLEL) triblock co-polymer. These polymers are listed in the GRAS category by the US FDA and have been used as a pharmaceutical excipient in a variety of formulations. The release of the exosomes from the triblock co-polymer assumed first-order kinetics, with over two weeks of steady release. This information was derived via a Transwell system through monitoring the number of exosomes. Due to this, the exosomes were administered weekly. Another study conducted by Thomas and colleagues was in favor of the use of semi-solid formulation to deliver the exosomes [193]. To demonstrate cartilage repair in C57BL/6 mice, exosomes were dispersed in liquid rat type I collagen gel. The result showed that a single dose of WNT3a-loaded exosomes improved the repair of osteochondral defects by activating the canonical WNT signaling pathway.

Apart from the use of synthetic polymers and collagen as exosome excipients, the use of hyaluronic acid was also tested. The report published in 2020 by Wong et al. used formulated exosomes with a HA mixture and tested it on osteochondral-defect-induced rabbits [194]. The administration was performed via intra-articular injection. Although the result of the study indicates a significant improvement in the macroscopical and histological examination, the experimental design did not include a control sample with exosomes alone. Hence, a comparison to the use of hyaluronic acid in assisting exosomes to elicit their responses could not be established. Nevertheless, a repaired layer of tissues, composed primarily of hyaline cartilage, was detected in the animals treated with exosomes and hyaluronic acid.

The use of exosomes in treating OA has been previously reviewed [195]. An intra-articular injection of exosomes to the side of the knee injury is favorable because of its ability to increase exosome accumulation at the target tissues and simultaneously reduce unnecessary systemic exposure. In this section, we highlight several recently conducted studies that have formulated exosomes with excipients to achieve efficient delivery. These excipients serve the eventual purpose of improving rheological properties and holding the exosomes at the injection site, thereby retaining high tissue exposure. The strategy is rationalized based on the physiological process (such as lymphatic drainage) that could potentially reduce the number of exosomes at the tissue vicinity. In addition, the selected studies in this section also used biomaterial excipients that are biocompatible. The objective—to minimize alteration in the exosomes—should be one of the priorities when designing a formulation to achieve effective exosome delivery.

### 8.2. Direct Needle Injection: Exosome Suspension

In general, most studies have utilized formulations with excipients to deliver the exosomes. However, there are also many studies that have reportedly injected the exosomes directly without much complex formulation. Liao and colleagues suggested that exosomes may not always need to be formulated, but, rather, they could be directly injected [196]. The exosomes were derived from bone marrow stem cells, isolated, characterized, and injected via the intra-articular route. The treatment plan involved twice-weekly injection for 4 weeks and was able to achieve reduced inflammation in the joints of experimental rats.

In a separate study, exosomes without complex formulation were administered to determine chondrocyte anabolism and articular cartilage regeneration [197]. Researchers injected an exosome suspension intra-articularly into New Zealand white rabbits that were artificially induced with cartilage injury (osteochondral defect). Although no injection frequency was mentioned, the macroscopic result was significant. Even with only 4 weeks duration of treatment, exosomes were able to induce the formation of a small number of cartilage-like structures in the defect area. When compared to the control group receiving only saline, the result showed that exosome dispersion could elicit pharmacological responses even without complex formulation.

The evaluation of the efficacy of exosomes in treating osteochondral defects also extends to the rodent model. Osteochondral defects were created on the trochlear grooves of the distal femurs of 8-week-old Sprague–Dawley rats [104]. Similar to the results reported by Shao et al. [194], a significant improvement compared to the negative control (PBS) was noted. The exosomes were administered weekly. By 12 weeks, the exosome-treated defects showed a complete restoration of cartilage and subchondral bone. In contrast, rats treated with PBS only displayed fibrous repair tissues at the defective site. These observations were confirmed by gross appearance and histological evaluation.

Due to the promising result of exosomes, Zhu et al. initiated a study to compare the efficacy of exosomes derived from different sources (induced pluripotent stem cell-derived MSC (iMSC) and synovial membrane-derived MSC (sMSC)) in treating OA [198]. The comparison was performed on the collagenase-induced OA mice model. In terms of the formulation, the exosomes were suspended in PBS before being injected intra-articularly on a weekly basis. The authors claimed that iMSC exosomes outperformed sMSC exosomes based on macroscopic and histological examinations. These superior effects of iMSC-derived exosomes were also documented in chondrocyte migration and proliferation. In fact, iMSC-derived exosomes can be procured more readily compared to sMSC-derived exosomes, which further supports the feasibility of using iMSC-derived exosomes for therapeutic purposes in the future.

The use of exosomes in an animal model can be achieved with or without complex formulations. From the perspective of formulation scientists, excipients are typically added to preserve the stability of the samples. If the concerns over the preservation of the exosomes could be achieved without additional excipients (such as antimicrobials), then the sample should be prepared with minimal alteration. Hence, the interaction of excipients with the exosomes, either chemically or pharmacologically, can be avoided. However, it is hypothesized that the exosomes suspended in PBS alone would require a higher frequency of administration compared to those stabilized in gel due to lymphatic drainage and the dynamics of body fluid movement.

## 9. Conclusions and Perspective

Although MSC-derived exosomes have shown great potential in protecting and repairing damaged cartilage, there is still much research to be done as information about the safety, efficacy, and mechanism of action of this novel therapeutic approach is still lacking. Specifically, the ideal cell source and the optimal dosage of exosomes for the treatment of cartilage damage remain unclear. In addition, enrichment of exosomes with specific miRNAs has shown promising results in cartilage repair in vitro and in vivo. This is another area that requires more exploration to identify the miRNA to be enriched in the exosomes for optimal results.

The method of exosome delivery to the OA joints is also under intense investigation. Research is ongoing to identify suitable biomaterials or scaffolds for more efficient delivery of the exosomes. The biomaterials should be able to protect and preserve the exosomes as this is important to maintain the integrity of the cargo. Encapsulation within biomaterials also permits the sustained delivery of exosomes for an elongated period. This is critical as exosomes have a short half-life in vivo, and multiple injections might be needed to achieve the desired therapeutic results. Multiple injections are associated with disadvantages, including being more time-consuming, costly, and tedious, causing more discomfort, and having poorer patient compliance. In the future, multiple injections can be avoided when persistent delivery is accomplished with the assistance of biomaterials.

Nowadays, researchers are also exploring the surface modification of exosomal membranes for more efficient uptake of the exosomes by the chondrocytes. This is important to impart additional functionality to the exosomes to ensure targeted uptake by the cells of interest. To make the situation more complicated, the cargo of exosomes is affected by the culture environment, and the purity of exosomes is affected by the isolation/purification method used. Thus, identification and potency testing are very important for the quality control and production of exosomes for clinical applications. Nonetheless, the development of potency assays is challenging due to the complex mechanism of action of exosomes. The International Society for Cell and Gene Therapy (ISCT) Exosomes Scientific Committee advocated that the potency assays should be able to quantify the attributes of exosomes that are essential for the anticipated biological activity of exosome therapy in specific diseases [199].

In summary, exosomes contain miRNAs that can modulate cartilage repair and regeneration by enhancing proliferation, attenuating apoptosis, promoting chondrogenesis, increasing cartilage matrix secretion, and subsiding inflammation. Thus, exosome therapy has great potential for treating cartilage diseases and promoting cartilage regeneration, especially after the limitations mentioned above are adequately addressed.

## Figures and Tables

**Table 1:** List of miRNAs in MSC-derived exosomes related to chondrocytes and cartilage regeneration.

miRNA	Target	Physiological Role	Ref.
miR-23b	PKA	Induce chondrogenic differentiation of human MSCs by inhibiting PKA signaling	[143]
miR-92a	Noggin3	Targets Noggin3 and activates the PI3K/Akt/mTOR pathway to positively regulate the proliferation and matrix synthesis of chondroprogenitors	[144] [145]
miR-125b	ADAMTS-4	miR-125b overexpression suppresses IL-1-induced upregulation of ADAMTS-4 in human OA chondrocytes	[146]
miR-320	MMP-13	Downregulates MMP-13 expression in both the ATDC5 cell model of chondrogenesis and IL-1-treated primary mouse chondrocytes	[147]
miR-145	Sox9	miR-145 inhibition upregulates Sox9 expression and promotes MSC chondrogenesis	[148]
miR-221	MDM2	Downregulates MDM2 to prevent slug protein degradation that, in turn, negatively regulates chondroprogenitor proliferation	[149]
miR-22	PPARA, BMP-7	miR-22 inhibition upregulates BMP-7 and PPARA expression, inhibits IL-1 expression, and suppresses MMP-13 expression in OA chondrocytes	[150]
miR-92a-3p	Wnt5a	Regulate cartilage development and homeostasis by targeting Wnt5a	[151]
miR-135b	Sp1a	Promote chondrocyte proliferation and cartilage repair in OA by downregulating Sp1a in chondrocytes	[152]
miR-100-5p	mTOR	Inhibit mTOR signaling pathway to enhance chondrocyte autophagy	[153]
miR-140-5p	YAP	Enhance ECM secretion and induce proliferation and migration of articular chondrocytes via activating YAP as well as prevent osteoarthritic joint damage	[154]
miR-26a-5p	PTGS2	Promote the survival of synovial fibroblasts and reduce synovitis	[155]
miR-136-5p	ELF3	Inhibit cartilage degeneration in traumatic osteoarthritis	[156]
miR-127-3p	CDH11-mediated Wnt/β-catenin pathway	Inhibit CDH11, thereby blocking the Wnt/β-catenin pathway in chondrocytes and reducing the chondrocyte damage in osteoarthritic joints	[157]
miR-9-5p	Syndecan-1	Has anti-inflammatory and cartilage protective effects on osteoarthritis	[158]

## Data Availability

Data sharing not applicable.
